# Genomewide association study for onset age in Parkinson disease

**DOI:** 10.1186/1471-2350-10-98

**Published:** 2009-09-22

**Authors:** Jeanne C Latourelle, Nathan Pankratz, Alexandra Dumitriu, Jemma B Wilk, Stefano Goldwurm, Gianni Pezzoli, Claudio B Mariani, Anita L DeStefano, Cheryl Halter, James F Gusella, William C Nichols, Richard H Myers, Tatiana Foroud, the PSG -PROGENI and GenePD Investigators, Coordinators and Molecular Genetic Laboratories

**Affiliations:** 1 Boston University School of Medicine, Boston, MA, USA; 2 Indiana University School of Medicine, Indianapolis, IN, USA; 3 Parkinson Institute, Istituti Clinici di Perfezionamento, Milano, Italy; 4 Boston University School of Public Health, Boston, MA, USA; 5 Massachusetts General Hospital and Harvard Medical School, Boston, MA, USA; 6 Cincinnati Children's Hospital Medical Center and University of Cincinnati College of Medicine, Cincinnati, Ohio, USA

## Abstract

**Background:**

Age at onset in Parkinson disease (PD) is a highly heritable quantitative trait for which a significant genetic influence is supported by multiple segregation analyses. Because genes associated with onset age may represent invaluable therapeutic targets to delay the disease, we sought to identify such genetic modifiers using a genomewide association study in familial PD. There have been previous genomewide association studies (GWAS) to identify genes influencing PD susceptibility, but this is the first to identify genes contributing to the variation in onset age.

**Methods:**

Initial analyses were performed using genotypes generated with the Illumina HumanCNV370Duo array in a sample of 857 unrelated, familial PD cases. Subsequently, a meta-analysis of imputed SNPs was performed combining the familial PD data with that from a previous GWAS of 440 idiopathic PD cases. The SNPs from the meta-analysis with the lowest p-values and consistency in the direction of effect for onset age were then genotyped in a replication sample of 747 idiopathic PD cases from the Parkinson Institute Biobank of Milan, Italy.

**Results:**

Meta-analysis across the three studies detected consistent association (p < 1 × 10^-5^) with five SNPs, none of which reached genomewide significance. On chromosome 11, the SNP with the lowest p-value (rs10767971; p = 5.4 × 10^-7^) lies between the genes *QSER1 *and *PRRG4*. Near the PARK3 linkage region on chromosome 2p13, association was observed with a SNP (rs7577851; p = 8.7 × 10^-6^) which lies in an intron of the *AAK1 *gene. This gene is closely related to *GAK*, identified as a possible PD susceptibility gene in the GWAS of the familial PD cases.

**Conclusion:**

Taken together, these results suggest an influence of genes involved in endocytosis and lysosomal sorting in PD pathogenesis.

## Background

Parkinson disease (PD), the second most common neurodegenerative disorder, is characterized by debilitating symptoms of tremor, rigidity, and bradykinesia, usually occurring late in life. PD incidence increases with age from 1.7/10,000 person-years between ages 50 to 59 to 9.3/10,000 person-years between ages 70 to 79 and has a prevalence of approximately 1.8% among people over the age of 65 [1]. While the average age of onset of PD is approximately 60 years, there is wide variation, with some individuals having onset before age 20 and others not until after age 90 [2,3].

Five monogenic forms of PD have been identified and characterized by mode of transmission, onset age and protein affected by mutation. These include α-synuclein (*SNCA *or PARK1) [4], parkin (PARK2) [5], PTEN-induced putative kinase 1 (*PINK1 *or PARK6) [6], *DJ-1 *(PARK7) [7], and leucine-rich repeat kinase 2 (*LRRK2 *or PARK8) [8]. Onset for PARK1 is younger than that seen for idiopathic PD [4]. PARK2 (parkin) is a recessive form with young onset, commonly before age 40. Heterozygous mutations in parkin are also associated with earlier onset of PD, typically in the early to mid sixth decade [9,10]. By contrast, PD associated with *LRRK2 *mutations presents an onset distribution very similar to that seen in idiopathic PD, as well as clear age-dependent penetrance [11-13].

Onset of PD has been shown to be correlated between siblings with PD [14] suggesting that genetic modifiers influence onset age. Segregation analyses in three independent studies showed evidence of a genetic effect influencing age of onset of PD [15-17]. Notably, all three of these segregation analyses showed stronger evidence for the presence of "major genes" influencing onset age or penetrance, than for genes influencing susceptibility. Furthermore, age is one of the strongest risk factors for PD, suggesting that age related penetrance is strongly associated with disease expression. By identifying genes related to onset age, it may be possible to identify pathogenic mechanisms and therapeutic targets capable of delaying onset of disease symptoms. Effectively postponing disease onset will reduce disease prevalence and ease the burden of PD in our aging population.

All prior PD genome wide association studies (GWAS) have focused exclusively on the detection of susceptibility genes and none has investigated association to genes influencing onset age [18-20]. In this study, we describe the first GWAS of onset age. This GWAS included 857 PD cases with a positive family history of PD. In addition, we performed a second GWAS with onset age as the phenotype using publicly available data from 440 randomly ascertained PD cases [19]. We conducted a meta-analysis of the two studies comprising approximately 2 million SNPs imputed using HapMap data. Finally, a replication study of the top findings from the meta-analysis was performed in an independent sample of 747 randomly ascertained PD cases from Milan, Italy.

## Methods

### PD Cases

One PD case (n = 935) from each family recruited from two ongoing studies of familial PD, the GenePD study and the PROGENI study, was selected for the GWAS. Both studies recruited families consisting of at least two members meeting diagnostic criteria for PD. PD cases underwent a uniform neurological evaluation that employed PD diagnostic criteria based on a modified version of the United Kingdom PD Society Brain Bank Criteria [21]. Detailed descriptions of the inclusion and exclusion criteria are described elsewhere for the PROGENI [22] and GenePD [14] studies. The analyzed sample was exclusively white, non-Hispanic.

No subject known to have a disease-producing mutation was included in this analysis. All cases were known to be negative for the *LRRK2 *G2019S mutation, and many, but not all, were also screened for PARK1(*SNCA*)(N screened = 702), PARK2 (*parkin*)(N = 593), PARK7 (*DJ1*)(N = 328), and *NR4A2 *(N = 550) [9,10,12,23-27]. PD onset age was determined by interview and reflected the age of first symptom of PD, which commonly preceded age of physician diagnosis. The reliability of ascertaining age of onset though interview compared to medical records has been estimated as 0.94 [28].

In addition, control samples (n = 895) obtained from the NINDS Human Genetics Resource Center DNA and Cell Line Repository (Camden, NJ) were intermixed on the same plates as the cases when genotyped. All selected control samples were reported to be white, non-Hispanic. While not used in the analysis of onset age of PD, these samples were used for SNP and sample quality assessment and genotype imputation as described below.

Appropriate written informed consent was obtained for all samples included in this study.

### Microarray Genotyping and Quality Assessment

Genotyping was performed by the Center for Inherited Disease Research (CIDR) using the Illumina HumanCNV370 version1_C BeadChips (Illumina, San Diego, CA, USA) and the Illumina Infinium II assay protocol [29]. As previously described [20], 78 cases and 28 controls were removed due to a low genotype call rate (<98% of SNPs), cryptic relatives in the sample, or population stratification. The final sample included 857 PD cases (all derived from whole blood) and 867 control DNA samples (all derived from lymphoblastoid cell lines). Genotype calls and quality scores were determined from allele cluster definitions for each SNP as determined by the Illumina BeadStudio Genotyping Module version 3.1.14 and the combined intensity data from 96% of study samples as previously described [20]. Genotype calls with a quality score (Gencall value) of 0.25 or higher were considered acceptable. Blind duplicate reproducibility was 99.98%. Although we performed additional SNP filtering described below, the CIDR released dataset contained 344,301 SNPs and is available on dbGaP (; Accession number: phs000126.v1.p1).

SNPs were removed if: 1) the call rate of the SNPs was lower than 98% (n = 7,764), 2) the minor allele frequency was less than 0.01 in the combined case and control dataset (n = 7,667), 3) there were differential rates of missing genotypes in the cases and controls (n = 75) or males and females (n = 271), or 4) significant deviation from Hardy Weinberg equilibrium was observed in the control sample (n = 906). Many markers failed multiple tests. The final dataset consisted of 328,189 SNPs that passed all quality control measures (94.6% of all attempted SNPs).

### Mayo-Perlegen LEAPS sample

Publicly available SNP genotype data for 443 PD cases was accessed from dbGaP for the GWAS "Mayo-Perlegen LEAPS (Linked Efforts to Accelerate Parkinson's Solutions) Collaboration" (; Accession number: phs000048.v1.p1; [19]. This data set included genotyping for the 198,345 SNPs meeting the quality control standards described by Maraganore et al. [19]. We examined these data for population stratification and for cryptic relatedness. While no population outliers were observed, three individuals were removed due to apparent second-degree relationships identified during our review of the data, leaving a sample of 440 PD cases included in these analyses. While data are also publicly available for 267 PD cases from the Fung et al. GWAS [18], that study only included cases whose age of PD onset was 55 years or greater resulting in an age distribution that was significantly different from the other two samples (p < 0.0001). Therefore, we did not include the cases from that study in our meta-analysis due to limited variability in the onset distribution.

### Population stratification

Both the GenePD-PROGENI and Mayo-Perlegen LEAPS sample sets were screened for population outliers during initial QC. In addition, after the final study sample sets were determined, principle components were recalculated independently for each study using only the samples included in the final analyses. Association to onset age was then tested for the first six principle components for each study. No association (p < 0.05) between the first six principle components and onset age was seen in either study sample.

### Imputation

Imputation was performed to increase the power of the meta-analysis and to facilitate the joint analysis of results generated from Mayo-Perlegen LEAPS and our GWAS, which were genotyped on different platforms. The program MACH 1.0 (compiled using Intel's optimized compiler) was used to impute genotypes for 2,543,887 autosomal SNPs characterized in the HapMap project [30-32].

The GenePD-PROGENI sample and the Mayo-Perlegen LEAPS cohort were imputed separately using phased haplotype data downloaded from the HapMap project website [33].

In the GenePD-PROGENI sample, imputation was performed using both cases and controls. A subset of 200 cases and controls with high call rates was selected to perform the initial model parameter calculation. Next, imputation was performed on all participants using all autosomal SNPs where the strand was not ambiguous (i.e. not an A/T or G/C SNP) and that passed all other quality control measures described previously. In the Mayo-Perlegen LEAPS cohort, all PD cases were used to compute the initial model parameters. All unambiguous autosomal SNPs passing quality review, as described in the original Mayo-Perlegen LEAPS GWAS [19], were used for this imputation. In addition, because the genotyping platform used in the Mayo-Perlegen LEAPS study included a high percentage of ambiguous SNPs, those ambiguous SNPs which could be confidently matched to HapMap strands through the comparison of minor allele frequencies were also used for imputation. For each study, initial model parameters were calculated using 100 iterations. SNP quality was assessed using the Rsq metric, which estimates the squared correlation between imputed and actual genotypes with SNPs having an Rsq <0.3 excluded from further study (N = 61,271).

At the time of this study, only autosomal imputation is supported by MACH; therefore, the program IMPUTE was used to impute SNPs on the X chromosome [34,35]. Haplotype, legend, and recombination rate files based upon HapMap (rel#21-NCBI build 35) were downloaded from IMPUTE's website for use in imputing 64,621 SNPs in the non-pseudoautosomal region of the X chromosome. Imputation was run in all participants in the GenePD-PROGENI study and separately in all PD cases in the Mayo-Perlegen LEAPS GWAS, using all X-chromosome SNPs that passed quality assessment as described above and were found in the legend file provided by IMPUTE.

### Statistical Analyses

In order to meta-analyze results from both studies, association to onset age was performed using exclusively imputed, not genotyped data, for 1,861,750 SNPs passing imputation QC and with minor allele frequencies greater than 10%. Imputed SNPs with minor allele frequencies less than 10% in the GenePD-PROGENI sample (N = 620,866) were excluded from the association analyses to avoid false positives that can occur in the analysis of low minor allele frequency SNPs. To evaluate SNP association under an additive mode of inheritance, the predicted allele dosage for each genotype estimated by MACH for autosomes or by IMPUTE for the X chromosome was used. To model the recessive and dominant modes of inheritance, the genotype probabilities calculated by MACH were used. While a combination of additive and recessive models can have high power to detect dominant effects, this power decreases as the minor allele frequency of the SNP nears 0.5 [36]. Therefore, we studied additive, dominant and recessive models. The total probability of having either one copy or two copies of the minor allele was used for the dominant model and the probability of having two copies of the minor allele was used for the recessive model. Recessive and dominant models were not studied for the X chromosome. Linear regression analyses were performed using SAS v9.1.

### Meta-Analysis

Since the genotypic data was generated on different arrays (Illumina and Perlegen) with few SNPs in common, we employed a conservative meta-analytic approach to combine results from the two studies. Meta-analysis of the results of the linear regression of the imputed data for the GenePD-PROGENI sample and the Mayo-Perlegen LEAPS cohort was performed using METAL . As is common in GWAS meta-analyses, a fixed effects model with standard error weighting was used, as random-effects models may be too conservative in GWA studies with a small number of studies[37].

### Replication Study

In order to validate the top findings from the GWAS meta-analysis, an additional sample of 896 PD cases with reported ages of PD onset was provided by the Parkinson Institute - Istituti Clinici di Perfezionamento, Milan, Italy from the "Human Genetic Bank of Patients Affected by PD and Parkinsonisms". These cases were recruited irrespective of family history or onset age, and similar to the GenePD-PROGENI, used the UK Parkinson's Disease Society Brain Bank criteria to confirm idiopathic PD [38] and defined onset as the age of first symptom of PD.

Twenty-four SNPs were selected based upon the following criteria (1) a p-value less than 0.00001 in the meta-analysis of the two GWAS, with (2) a consistent direction of effect in both studies. Nine SNPs meeting these criteria were identified from the additive inheritance model, thirteen from the dominant model and eleven from the recessive model. Four SNPs were identified in both the additive and dominant model, one SNP was identified in both the additive and recessive model and one SNP was identified by all three models. Three gene regions were identified under two different genetic models with different SNPs. For each multiply-nominated gene region, the SNP from the model with the smaller p-value was selected for replication. These SNPs were genotyped using TaqMan technology implemented on the ABI PRISM^® ^7900HT Sequence Detection system (Applied Biosystems: Foster City, CA) at Boston University School of Medicine. Individual samples (149) with genotyping call rates of less than 95% were excluded from further analysis.

Association of the 24 SNPs to onset age was evaluated in the 747 remaining Italian cases using linear regression performed with the software Plink v1.01 [39] using the corresponding genetic model (additive, recessive or dominant) by which each SNP was originally identified. A final fixed effects meta-analysis of all three studies was performed using METAL.

To distinguish whether associations observed were to age in general, as opposed to age at onset of PD, linear regression to censoring age was performed in the 867 NINDS control samples genotyped with the GenePD-PROGENI cases.

## Results

Demographic characteristics of the three samples studied are shown in Table 1. All three studies have a similar percentage of male participants. The GenePD-PROGENI and Mayo-Perlegen LEAPS samples have similar mean ages of PD onset, while the Italian sample has a somewhat younger average age at onset. The GenePD-PROGENI sample has the widest range of onset ages from 19 to 90 years while the Mayo-Perlegen LEAPS has no participants under 30 years of age and the Italian sample has no participants over 81 years of age. No significant differences in onset age are seen between men and women in any of the studies.

**Table 1:** Age and sex distribution of the GenePD- PROGENI, Mayo-Perlegen LEAPS, and Milan, Italian study samples.

	**N**	**% Male**	**Mean Onset**	**Std Dev Onset**	**Range Onset**
GenePD-PROGENI	857	59.2	61.9	10.9	19-90

Mayo-Perlegen LEAPS	440	61.6	61.0	11.2	30-94

Milan, Italian	747	59.4	55.2	10.8	20-81

**Figure 1:**
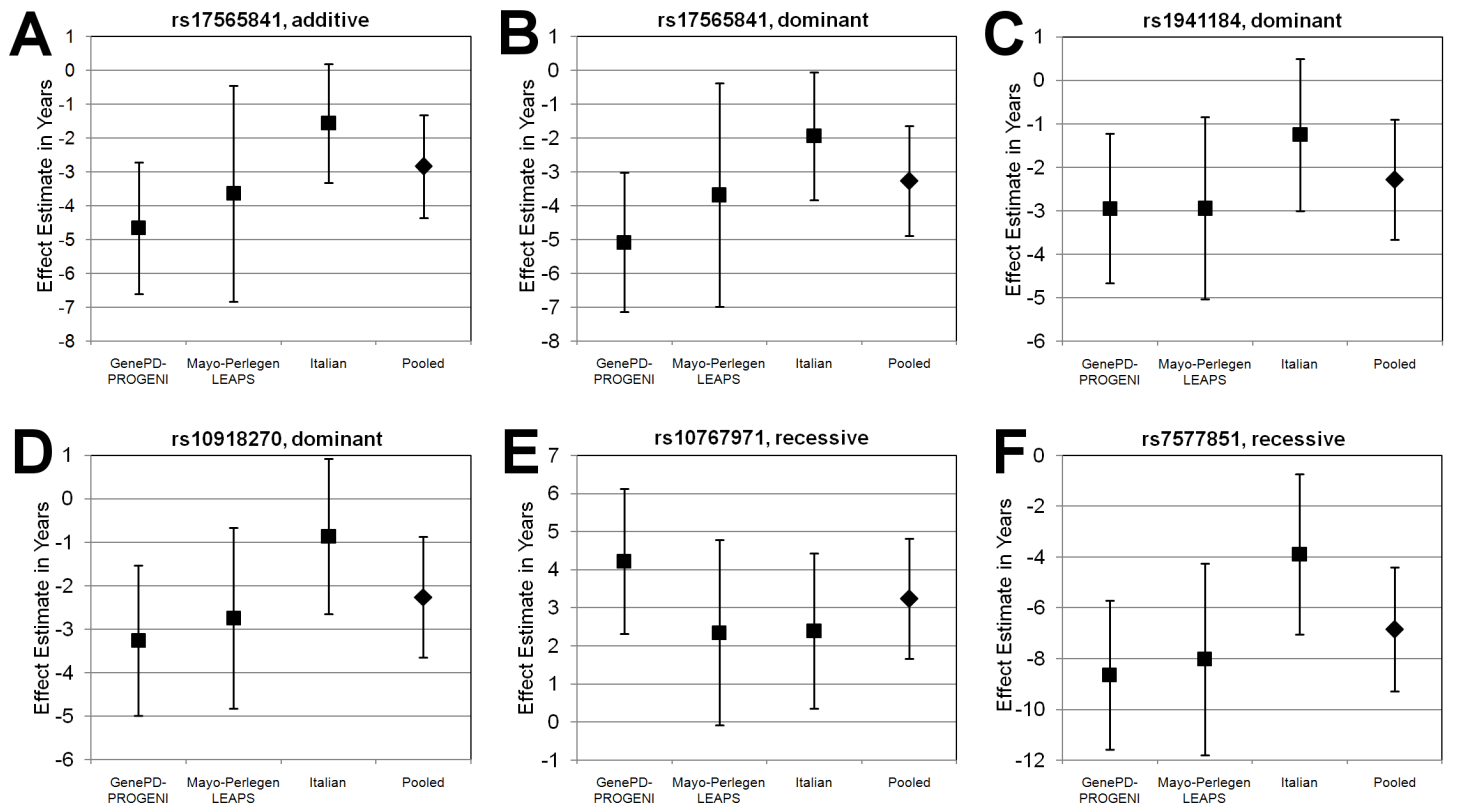
**Forest plots showing study-specific and pooled effects for top six results in final meta-analysis**. The effect (in years) on age at PD onset and 95% confidence intervals for the indicated genetic model are shown for each of the three independent samples and the combined analysis. **A**. Additive model of the A allele in SNP rs17565841 **B**. Dominant model of the A allele in SNP rs17565841 **C**. Dominant model of the C allele in SNP rs1941184 **D**. Dominant model of the A allele in SNP rs10918270 **E**. Recessive model of the T allele in SNP rs10767971 **F**. Recessive model of the T allele in SNP rs7577851.

Supplementary Tables S1 (additive model), S2 (dominant model) and S3 (recessive model) (see Additional file 1) present the top SNPs from each region with a meta-analysis p < 0.0001 for imputed SNP data in the GenePD-PROGENI and Mayo-Perlegen LEAPS studies. Twenty-four SNPs with meta-analysis p-values of less than 0.00001 and with a consistent direction of effect for both GWAS were genotyped in the Italian replication sample of 747 PD cases (Table 2). SNPs genotyped in either the GenePD-PROGENI or Mayo-Perlegen LEAPS GWAS platforms are distinguished by notation from those SNPs imputed by both studies. The results of the association analysis in the Italian sample, as well as the combined meta-analysis of all three samples, are shown in Tables 3 (additive), 4 (dominant), and 5 (recessive) and in Figure 1.

**Table 2:** Location and minor allele frequency (MAF) information for 24 SNPs selected for replication study in Italian sample

**Chr**	**SNP**	**SNP position**^a^	**Minor Allele**	**MAF**	**Genes in Region**^b^
1	rs7556447	2,335,959	G	0.24	*PEX10*

1	rs1355637^c^	148,849,062	T	0.19	*MCL1|ENSA*

1	rs10918270	160,182,125	A	0.41	*ATF6*

2	rs11899121	20,231,454	C	0.50	*SDC1*

2	rs7577851^c^	69,577,214	T	0.17	*AAK1*

2	rs17817190	134,068,118	G	0.11	*NAP5*

6	rs6936388	42,275,464	T	0.25	*CCND3|TAF8|GUCA1A|GUCA1B|MRPS10*

6	rs1572662	154,853,289	A	0.36	*CNKSR3|CLDN20|RBM16|TIAM2|TFB1M*

7	rs1420143^c^	29,514,849	C	0.44	*CHN2*

7	rs17663983	76,243,177	T	0.12	

10	rs12261736	52,738,610	T	0.28	*PRKG1*

10	rs7076519	127,738,829	C	0.33	*ADAM12*

11	rs10767971^c^	32,852,240	T	0.42	*PRRG4|QSER1*

12	rs12829697	30,067,223	G	0.13	

12	rs10773917	130,572,784	T	0.23	

13	rs4771006^d^	26,180,684	A	0.49	*WASF3*

13	rs676495^c^	29,561,754	T	0.24	

14	rs10147486	47,054,769	T	0.27	*MDGA2*

15	rs17565841	25,670,842	A	0.10	*OCA2*

17	rs9904572	12,803,084	A	0.34	*RICH2*

17	rs4791571^c^	14,155,801	A	0.42	*HS3ST3B1*

18	rs1941184	27,292,121	C	0.30	*DSG3*

19	rs10420134	40,953,768	G	0.18	*C19orf55|SNX26*

20	rs3887942	23,925,072	G	0.19	*GGTLC1*

^a ^From NCBI Build 36 reference; ^b ^Nearest genes within 100 kb of SNP;^c ^SNP was genotyped in Mayo-Perlegen LEAPS GWAS;^d ^SNP was genotyped in GenePD-PROGENI GWAS

**Table 3:** Additive model study-specific and meta-analysis results

	**GenePD-PROGENI**		**Mayo-Perlegen LEAPS**		**GWAS Meta-analysis**			**Milan, Italian**		**Three Sample Meta-analysis**		
**SNP**	**effect**	**p-value**	**effect**	**p-value**	**Direction of Effect**	**effect**	**p-value**	**effect**	**p-value**	**Direction of Effect**	**effect**	**p-value**

rs17565841	-4.66	2.7 × 10^-6^	-3.64	1.7 × 10^-1^	--	-4.53	9.1 × 10^-7^	-1.57	5.0 × 10^-2^	---	-2.84	2.6 × 10^-6^

rs10918270	-2.12	1.1 × 10^-4^	-1.90	1.6 × 10^-2^	--	-2.05	4.8 × 10^-6^	-0.62	2.7 × 10^-1^	---	-1.49	2.0 × 10^-5^

rs12261736	-3.61	1.8 × 10^-7^	-1.17	4.0 × 10^-1^	--	-3.13	3.5 × 10^-7^	-0.42	4.5 × 10^-1^	---	-1.65	6.6 × 10^-5^

rs4771006	2.35	1.6 × 10^-3^	2.59	1.6 × 10^-3^	++	2.46	7.3 × 10^-6^	0.50	3.8 × 10^-1^	+++	1.52	1.2 × 10^-4^

rs1420143	2.08	1.4 × 10^-4^	2.58	8.1 × 10^-3^	++	2.20	3.6 × 10^-6^	0.02	9.8 × 10^-1^	+++	1.27	4.1 × 10^-4^

rs6936388	2.45	4.2 × 10^-4^	2.98	7.2 × 10^-3^	++	2.60	9.2 × 10^-6^	-0.31	7.0 × 10^-1^	++-	1.58	8.2 × 10^-4^

rs10147486	2.21	3.1 × 10^-4^	2.89	8.0 × 10^-4^	++	2.44	9.2 × 10^-7^	-0.69	3.0 × 10^-1^	++-	1.31	9.5 × 10^-4^

rs7076519	-2.71	9.3 × 10^-6^	-2.53	2.5 × 10^-2^	--	-2.67	6.0 × 10^-7^	0.68	1.8 × 10^-1^	--+	-0.92	1.3 × 10^-2^

**Table 4:** Dominant model study-specific and meta-analysis results

	**GenePD-PROGENI**		**Mayo-Perlegen LEAPS**		**GWAS Meta-analysis**			**Milan, Italian**		**Three Sample Meta-analysis**		
**SNP**	**effect**	**p-value**	**effect**	**p-value**	**Direction of Effect**	**effect**	**p-value**	**effect**	**p-value**	**Direction of Effect**	**effect**	**p-value**

rs17565841	-5.08	4.7 × 10^-6^	-3.68	2.0 × 10^-1^	--	-4.90	1.9 × 10^-6^	-1.94	3.6 × 10^-2^	---	-3.26	2.1 × 10^-6^

rs1941184	-2.94	1.3 × 10^-4^	-2.94	1.0 × 10^-2^	--	-2.94	3.7 × 10^-6^	-1.25	1.2 × 10^-1^	---	-2.28	4.3 × 10^-6^

rs10918270	-3.26	2.9 × 10^-5^	-2.74	1.5 × 10^-2^	--	-3.09	1.2 × 10^-6^	-0.87	2.9 × 10^-1^	---	-2.26	7.5 × 10^-6^

rs3887942	4.17	7.2 × 10^-4^	7.10	1.5 × 10^-3^	++	4.85	6.4 × 10^-6^	0.93	2.6 × 10^-1^	+++	2.37	2.7 × 10^-4^

rs12261736	-4.03	9.5 × 10^-6^	-2.04	2.8 × 10^-1^	--	-3.66	7.3 × 10^-6^	-0.46	5.7 × 10^-1^	---	-2.07	3.5 × 10^-4^

rs10773917	3.15	3.8 × 10^-5^	2.58	3.5 × 10^-2^	++	2.99	3.7 × 10^-6^	-0.13	8.7 × 10^-1^	++-	1.75	4.8 × 10^-4^

rs10420134	-3.21	5.8 × 10^-5^	-3.12	5.1 × 10^-2^	--	-3.19	7.1 × 10^-6^	0.56	5.1 × 10^-1^	--+	-1.67	2.3 × 10^-3^

rs9904572	3.57	4.3 × 10^-6^	2.72	6.4 × 10^-2^	++	3.38	7.2 × 10^-7^	-1.05	1.9 × 10^-1^	++-	1.55	3.1 × 10^-3^

rs17817190	-3.87	3.9 × 10^-5^	-5.27	2.1 × 10^-2^	--	-4.07	2.5 × 10^-6^	1.30	2.1 × 10^-1^	--+	-1.85	5.3 × 10^-3^

rs1572662	3.57	4.1 × 10^-5^	4.89	7.5 × 10^-2^	++	3.69	7.8 × 10^-6^	-0.67	4.1 × 10^-1^	++-	1.45	1.2 × 10^-2^

rs7076519	-3.73	1.2 × 10^-5^	-3.81	3.0 × 10^-2^	--	-3.74	9.3 × 10^-7^	1.48	6.2 × 10^-2^	--+	-1.23	2.6 × 10^-2^

**Table 5:** Recessive model study-specific and meta-analysis results

	**GenePD-PROGENI**		**Mayo-Perlegen LEAPS**		**GWAS Meta-analysis**			**Milan, Italian**		**Three Sample Meta-analysis**		
**SNP**	**effect**	**p-value**	**effect**	**p-value**	**Direction of Effect**	**effect**	**p-value**	**effect**	**p-value**	**Direction of Effect**	**effect**	**p-value**

rs10767971	4.23	9.3 × 10^-6^	2.35	1.3 × 10^-1^	++	3.71	4.3 × 10^-6^	2.40	2.6 × 10^-2^	+++	3.24	5.4 × 10^-7^

rs7577851	-8.64	1.2 × 10^-4^	-8.03	3.1 × 10^-2^	--	-8.48	9.8 × 10^-6^	-3.89	1.3 × 10^-1^	---	-6.85	8.7 × 10^-6^

rs1355637	-5.10	2.3 × 10^-3^	-14.25	4.1 × 10^-5^	--	-6.83	5.1 × 10^-6^	1.10	6.0 × 10^-1^	--+	-4.19	6.2 × 10^-4^

rs11899121	-3.41	8.0 × 10^-5^	-3.33	1.6 × 10^-2^	--	-3.39	3.5 × 10^-6^	0.43	6.5 × 10^-1^	--+	-1.99	6.3 × 10^-4^

rs12829697	-10.51	8.5 × 10^-5^	-10.33	3.0 × 10^-2^	--	-10.47	7.0 × 10^-6^	0.68	8.1 × 10^-1^	--+	-6.15	7.2 × 10^-4^

rs17663983	-16.72	1.2 × 10^-5^	-25.53	4.6 × 10^-1^	--	-16.83	8.1 × 10^-6^	5.77	2.9 × 10^-1^	--+	-9.46	2.3 × 10^-3^

rs12261736	-8.48	1.9 × 10^-6^	-0.56	8.9 × 10^-1^	--	-7.14	9.6 × 10^-6^	-0.73	4.9 × 10^-1^	---	-2.65	2.7 × 10^-3^

rs1420143	3.31	7.9 × 10^-4^	6.05	1.3 × 10^-3^	++	3.90	7.3 × 10^-6^	-0.52	5.9 × 10^-1^	++-	1.89	3.3 × 10^-3^

rs676495	-6.90	1.9 × 10^-5^	-11.27	2.4 × 10^-1^	--	-7.02	9.1 × 10^-6^	1.86	2.7 × 10^-1^	--+	-2.84	1.4 × 10^-2^

rs7556447	-13.34	2.1 × 10^-5^	-11.11	1.9 × 10^-1^	--	-13.08	7.9 × 10^-6^	-0.44	7.7 × 10^-1^	---	-3.01	2.3 × 10^-2^

rs4791571	-5.18	1.3 × 10^-5^	-6.39	2.4 × 10^-1^	--	-5.23	5.9 × 10^-6^	2.57	3.5 × 10^-2^	--+	-1.52	6.9 × 10^-2^

Although ten of the replication SNPs showed a consistent direction of effect across all three studies, only two SNPs (both of which were genotyped on the Mayo-Perlegen LEAPS GWAS platforms) resulted in increased statistical evidence of association when combining the three studies, and both of these were in a recessive model. The most strongly associated SNP, rs10767971, located on chromosome 11 between the genes *QSER1 *and *PRRG4*, was associated with a 3.2 year older PD onset in individuals with 2 copies of the minor allele (p = 5.4 × 10^-7 ^in the 3 sample meta-analysis, compared to 4.3 × 10^-6 ^in the 2 sample meta-analysis). Conversely, an estimated 6.9 year earlier age of onset (p = 8.7 × 10^-6 ^in the 3 sample meta-analysis compared to 9.8 × 10^-6 ^in the 2 sample meta-analysis) was observed for the SNP rs7577851, located in the 16^th ^intron of the gene *AAK1 *on chromosome 2.

The most highly associated SNP in both the additive and dominant three sample meta-analyses, rs17565841, is located approximately 3 kb from the 3' end of the gene *OCA2 *on chromosome 15. This SNP was associated with an average 2.8 years younger onset age (p = 2.6 × 10^-6^) under an additive model and a 3.3 years younger onset age (p = 2.1 × 10^-6^) under a dominant model. Inclusion of the Italian cases in the meta-analysis did not strengthen the evidence of association as compared to the results seen in the two sample meta-analyses (9.1 × 10^-7 ^for additive and 1.9 × 10^-6 ^for dominant). However, in the Italian replication sample, this SNP did provide modest statistical association to onset age (p = 0.05 for additive and p = 0.04 for dominant) with the same direction of effect seen in the two other studies.

Also showing consistent directions of effect and p-values in the three-sample meta-analysis at the level of p < 1 × 10^-5 ^were two SNPs located in the genes *DSG3 *and *ATF6*. The SNP rs1941184, had the second best p-value identified under the dominant model and is located in the third intron of the gene *DSG3 *on chromosome 18. This SNP was associated with an average 2.3 year younger age of onset of PD across the three studies (p = 4.3 × 10^-6^). The SNP rs10918270, located in the 15^th ^intron of the gene *ATF6 *on chromosome 1, was identified under both additive and dominant modes of inheritance, but showed stronger association in the three sample meta-analysis under the dominant model (p = 7.5 × 10^-6^) with an average 2.3 year younger onset of PD. No association to age with direction of effect consistent with that observed for onset age of PD was seen in the control sample for any of the 24 SNPs at a significance level equal to 0.05.

## Discussion

We present results from the first GWAS for age at onset of PD, including a meta-analysis with the publicly available Mayo-Perlegen LEAPS GWAS data (dbGaP Study Accession: phs000048.v1.p1) and a follow-up replication study in an independent PD sample recruited in Milan, Italy. Differences were observed in the age distributions in the three populations used in this study (Table 1). However, there were no imposed age restrictions in any of the studies and a wide distribution of ages was represented in all three populations, each with a range of greater than 60 years.

No SNP reached the commonly accepted criterion for genome-wide significance of p < 5 × 10^-8 ^[40], which is based on recent estimates of independent genomewide sequence variation to maintain 5% genomewide type I error rate [41,42]. While this criterion provides an appropriate cutoff for determining significance for the large number of SNPs provided by imputation, this measure does not account for the testing of multiple genetic models, as was performed in this study. Despite the lack of genomewide significance, the meta-analysis in this study showed evidence of several interesting loci with consistent effects on onset age of PD across the three independent populations studied.

**Figure 2:**
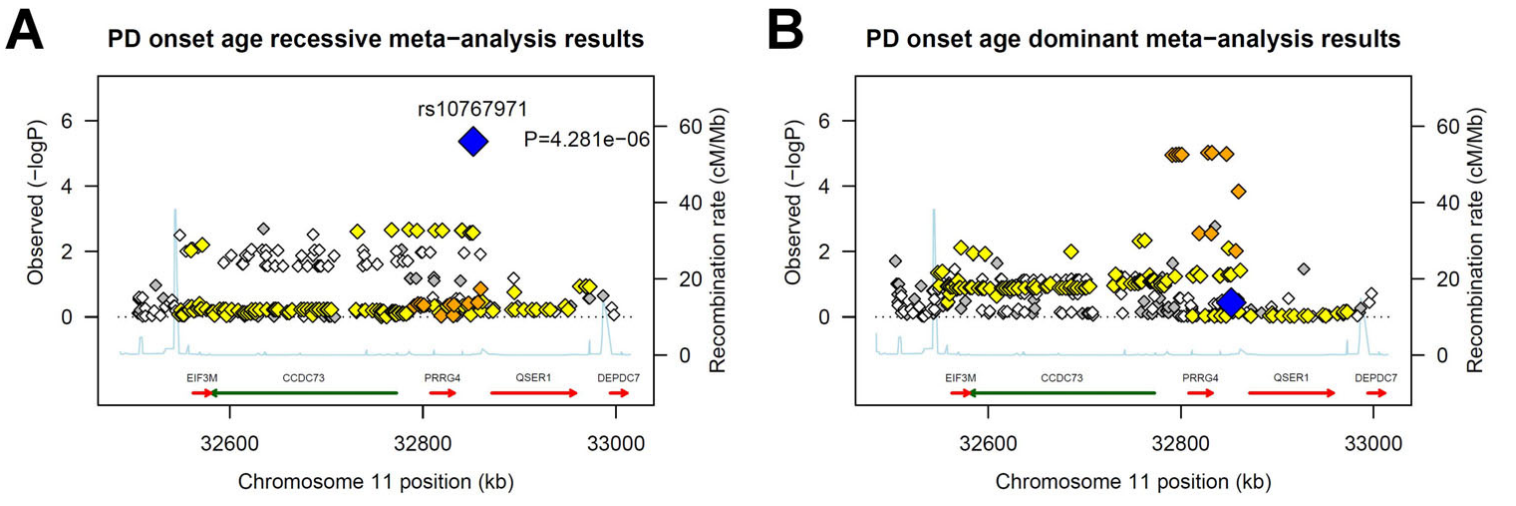
**Evidence of association in Chromosome 11 region**. Along the x axis is the physical position in the region (in kB) with known genes shown in their orientation The left Y-axis denotes the association test result as -log(p-value) corresponding to diamonds in the figure. The blue diamond identifies the primary SNP result labeled with an rs# and p-value. The color of additional diamonds depicts the pairwise linkage disequilibrium with the primary SNP: red indicates r^2 ^> 0.8, orange 0.5 < r^2 ^< 0.8, yellow 0.2 < r^2 ^< 0.5, white r^2 ^< 0.2, grey unknown LD. R^2 ^values were obtained from the CEPH HapMap data. The right Y-axis indicates the recombination rate, also obtained from CEPH HapMap data, and shown within the figure by the solid light-blue line. **A**. Recessive model **B**. Dominant model.

The SNP with the strongest evidence for association to onset age, rs10767971, is associated with a later age of onset under a recessive model. This SNP is nearly equidistant between the genes *PRRG4 *and *QSER1 *on chromosome 11, at just under 20 kb from each. The association results for nearby SNPs studied in the meta-analysis of the two GWAS as well as the LD structure and recombination rates for the region are shown in Figure 2A and 2B under recessive and dominant models. While the strongest evidence of association appeared with a recessive model, many nearby SNPs in strong LD showed similar association under the dominant model. The pattern suggests a single association, spanning several SNPs that may be more evident around the *PRRG4 *gene and may extend toward the *CCDC73 *and *EIF3M *genes.

The SNP showing the strongest association to PD onset across the three populations under both the additive and dominant models lies 3 kb from the 3' end of the gene *OCA2 *(oculocutaneous albinism II). Mutations in *OCA2 *are responsible for the most common form of albinism, oculocutaneous albinism type 2 [43] and several studies have shown this gene to be associated with common skin, hair and eye color variation found in European populations [43-48]. Variations in *OCA2 *have been associated with susceptibility to melanoma [49], which has also been reported to occur with increased frequency among PD cases [50-52]. Because of the targeted degradation of pigmented neurons in PD brains, these associations have lead to a hypothesized link between genes involved in pigmentation, such as *OCA2*, and PD, likely mediated through common elements in melanin and neuromelanin synthesis[53]. The protein encoded by *OCA2*, 'P' protein, is involved in the transport of tyrosine, a precursor to melanin, as well as in the regulation of melanosomal pH which may be key to the initiation of the enzyme controlling melanin synthesis in melanocytes [54]. It is not clear whether synthesis of neuromelanin is also regulated through melanosomal pH or is affected by the 'P' protein in a similar way [55]. Nevertheless, the association between *OCA2 *and younger age of onset are suggestive of a neuromelanin-related mechanism of effect.

The association of an intronic SNP in the gene *DSG3 *(desmoglein 3 (pemphigus vulgaris antigen)) may also be indicative of a neuromelanin related effect on onset age of PD. The protein encoded by *DSG3 *is the autoantigen for the autoimmune skin disease pemphigus vulgaris and this gene is expressed primarily in skin. Interestingly, some reports have demonstrated increased expression of *DSG3 *in melanocytes compared to keratinocytes (the most common cell type in the epidermis) [56].

An intronic SNP in the gene *ATF6 *also showed strong association to earlier age of onset of PD. *ATF6 *(activating transcription factor 6) transcribes a transcription factor localized to the endoplasmic reticulum (ER). The *ATF6 *protein is a critical regulator of the unfolded protein response (UPR), a highly conserved pathway activated in response to ER stress, and is a protective cellular response to the accumulation of misfolded proteins [57-59]. The UPR has been implicated in neurotoxin based cellular models of PD [60] and has also been shown to be activated by the over-expression of α-synuclein in yeast cells [61]. More recently, postmortem studies of PD case and control brains have shown activation of the UPR in cases, but not controls and that this activation is associated with the aggregation of α-synuclein [62].

The finding of an intronic SNP in *AAK1 *associated with PD onset age is intriguing because of the gene's genomic location, its function, and its close relation to a gene identified for PD susceptibility. The *AAK1 *(AP2 associated kinase 1) gene is located on chromosome 2p14, near 2p13, which has previously been implicated as the PARK3 locus[63], and for which linkage to onset age was demonstrated in both the GenePD [64] and PROGENI [65] studies. The *AAK1 *gene itself has not been previously implicated by positional mapping, but a microarray study of PD brain compared to controls demonstrated differential expression of *AAK1 *[66]. In our past GWAS of PD susceptibility in the GenePD-PROGENI cohort [20], the region containing the gene *GAK *(cyclin G-associated kinase) had the strongest evidence for association (chromosome 4p). The *AAK1 *and *GAK *genes both function at multiple steps in clathrin-mediated vesicular transport and the two kinases likely have some redundant functions [67] related to their homologous serine/threonine-kinase domain [68]. Recently, cathepsin D was implicated as the main lysosomal enzyme involved in α-synuclein degredation [69], and depletion of *GAK *was shown to impair the lysosomal sorting of cathepsin D [68]. Thus, the finding that *AAK1 *influences onset age and *GAK *influences risk in familial PD suggest that pathways involving lysosomal activity influence PD risk.

Several previous genome scans have provided evidence of loci linked to onset age, including 2p13 seen in both the GenePD and PROGENI studies, as mentioned above. Evidence of linkage has also been reported on chromosomes 1p, 1q, 8q, 9q, 10q, 20 and 21[64,65,70], but there is little overlap between these linkage regions and our top meta-analysis association results. Aside from the identification of the *AAK1 *gene near 2p13, the strongest association result observed under one of the previously reported linkage regions occurs near the gene sortilin-related VPS10 domain containing receptor 3 (*SORCS3*) located near the LOD score peak at 10q, originally identified in a combined linkage scan of onset age in PD and Alzheimer's disease [70]. This association is seen most strongly under a dominant model (p = 2.5 × 10^-5^) with minor allele carriers having an estimated older onset age by 3.3 years (see Additional file 1: Table S2).

A final region of interest is 15q26.2 that includes the gene *MCTP2*. Several SNPs in this region showed association to earlier onset age of PD in the meta-analysis of the two GWAS under the recessive model (see Additional file 1: Table S3). These SNPs did not reach the criteria for inclusion in the replication study, as the strongest p-value seen was 2.2 × 10^-5 ^with rs17504636. This SNP was associated with a 9.2 year earlier average PD onset. This region overlaps with a SNP reported in the susceptibility GWAS including these cases [20]. In that study the SNP rs4476132 was associated with PD susceptibility with an odds ratio of 1.3 (p = 7.7 × 10^-5^- meta-analysis with additive model). This overlap is consistent with a locus associated with a risk for younger-onset PD or with an effect modifying age dependent penetrance. The gene in this region, *MCTP2 *(multiple C2 domains, transmembrane 2), is expressed in the brain and has been implicated in linkage and association studies of abdominal fat [71] and major depression [72].

Important distinctions can be made between those genes that influence susceptibility for developing disease, and the genetic modifiers that influence penetrance or, as studied here, onset age. Perhaps the best examples for genetic modifiers are seen for Huntington's disease (HD) where an expanded CAG trinucleotide repeat on chromosome 4p16.3 causes the disease, but wide variation in onset age is evident for individuals with identical repeat lengths. The identification of the genes that presumably interact with huntingtin to produce relatively younger or older onset for a given repeat size provide insight into the pathogenic mechanisms for HD, as well as therapeutic targets for intervention [73-75]. Similarly, identifying those genes and their products that are associated with older onset in PD may provide insight into the disease mechanisms and processes for delaying onset with implications for novel treatments.

## Conclusion

The identification of the 15q26.2 region as well as the related genes *AAK1 *and *GAK *in the studies of PD onset age and susceptibility highlights the importance of the continued study of both of these traits, both separately and in combination. The direct overlap in affection and onset age association results in the 15q26.2 region shows this to be a candidate region that would benefit from further examination with consideration of important age-related effects, for example in studies correlating expression of genes in this region with onset age. The identification of association to onset age with the gene *AAK1*, in the same pathway as a previously identified susceptibility-associated gene *GAK *highlights the importance of genetic pathways in PD etiology, showing that the genes along the same pathway may have redundant effects or may modify disease pathology different ways, observed by differences in disease onset and progression. Studying PD in the context of onset age provides fundamental insight into the disease process and is essential to understanding mechanisms that modify disease penetrance and therefore may be key in identifying therapeutic targets.

## Competing interests

The authors declare that they have no competing interests.

## Authors' contributions

JCL participated in conception and design of the study, conducted statistical analyses, participated in the interpretation of data, and drafted the manuscript. NP, AD and JBW participated in the conception and design of the study, conducted statistical analyses, participated in the interpretation of data, and revised the article critically for important intellectual content. SG, GP, CBM, ALD, CH, JFG, WCN, RHM, and TF participated in the conception and design of the study, participated in the interpretation of data, and revised article critically for important intellectual content. All authors read and approved the final manuscript.

## Pre-publication history

The pre-publication history for this paper can be accessed here:



## Supplementary Material

Additional file 1 **Supplementary Tables**. The excel file includes three supplementary tables. Supplementary Table S1 includes regions showing associations at p < 0.0001 for the additive model GWAS meta-analysis and study-specific results. Supplementary Table S2 includes regions showing associations at p < 0.0001 for the dominant model GWAS meta-analysis and study-specific results. Supplementary Table S3 includes Regions showing associations at p < 0.0001 for the recessive model GWAS meta-analysis and study-specific results Click here for file
